# Suppressive and enhancing effects in early visual cortex during illusory shape perception: A comment on Kok and de Lange (2014)

**DOI:** 10.1068/i0689

**Published:** 2015-01-22

**Authors:** Pieter Moors

**Affiliations:** Laboratory of Experimental Psychology, University of Leuven (KU Leuven), Leuven, Belgium; e-mail: pieter.moors@ppw.kuleuven.be

**Keywords:** predictive coding, illusory shape perception, neural adaptation

## Abstract

In a recent functional magnetic resonance imaging study, Kok and de Lange (2014) observed that BOLD activity for a Kanizsa illusory shape stimulus, in which pacmen-like inducers elicit an illusory shape percept, was either enhanced or suppressed relative to a nonillusory control configuration depending on whether the spatial profile of BOLD activity in early visual cortex was related to the illusory shape or the inducers, respectively. The authors argued that these findings fit well with the predictive coding framework, because top-down predictions related to the illusory shape are not met with bottom-up sensory input and hence the feedforward error signal is enhanced. Conversely, for the inducing elements, there is a match between top-down predictions and input, leading to a decrease in error. Rather than invoking predictive coding as the explanatory framework, the suppressive effect related to the inducers might be caused by neural adaptation to perceptually stable input due to the trial sequence used in the experiment.

Popular theories of visual perception posit that the visual system is akin to an inference machine, applying (hierarchical) Bayesian inference to the bottom-up sensory signals to maximize the posterior probability of a prediction/hypothesis given the sensory signals (Clark, 2013; Gregory, 1980; von Helmholtz, 1860). One mechanism through which this inference process could work is predictive coding or free-energy minimization in which top-down predictions about the upcoming input are continuously generated (at multiple levels of the visual system), compared with the bottom-up sensory signal, and an error signal based on the difference between prediction and input is fed forward based upon which new predictions can be generated to further minimize prediction error. In this framework, feedback signals convey the predictions of the generative model, whereas feedforward connections transmit the discrepancy between prediction and sensory input (i.e., the error signal) (Friston, 2005; Mumford, 1992; Rao & Ballard, 1999). Depending on the (mis)match between prediction and bottom-up input, neural activity might be suppressed or enhanced in the case of a match or mismatch, respectively (Kok & de Lange, 2014).

In a recent study, Kok and de Lange (2014) sought to test these predictions using the well-known Kanizsa illusory shape stimulus (Figure 1A). In this stimulus, pacmen-like inducers give rise to an illusory shape percept, perceived as a foreground figure relative to the inducers in the background (Kanizsa, 1979; Kogo & Wagemans, 2013; Wagemans et al., 2012). Within the proposed framework, the authors predicted that neural activity in early visual cortex related to the inducers should go down, because top-down predictions match the bottom-up sensory input. However, the reverse should hold for neural activity related to the illusory shape. That is, the top-down predictions would signal a shape whereas none is to be found in the sensory signal.

**Figure 1.**
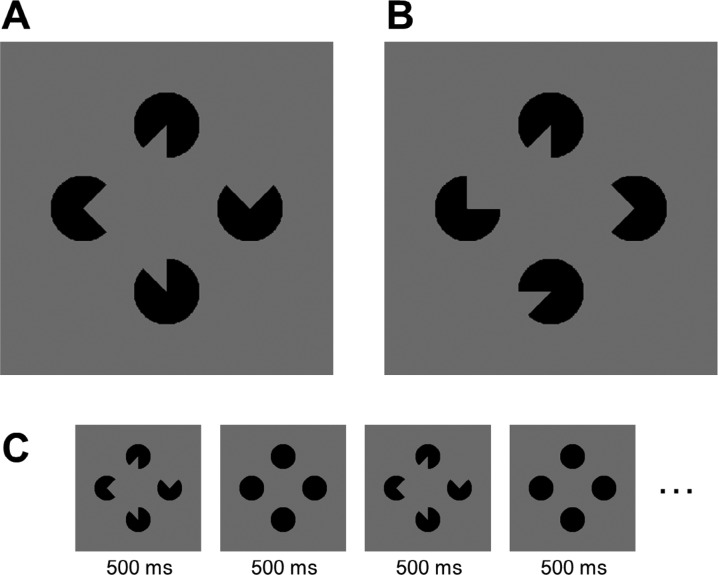
(A) Illusory shape stimulus example. (B) Control configuration example. (C) Trial sequence with alternating stimuli in the illusory shape condition. Note that this sequence does not depict the stimuli for the letter nor figure tasks.

Combining functional magnetic resonance imaging (fMRI) and population-receptive field (pRF) mapping (Dumoulin & Wandell, 2008), the authors delineated voxels in early visual cortex (V1 and V2) for which the estimated pRF fell in the region where either the inducers or the illusory shape was presented. As such, they obtained separate estimates for BOLD signal activation related to the inducer region and the illusory shape region.

In line with their predictions, Kok and de Lange (2014) show that, across two different tasks (controlling for attentional allocation to the stimuli), BOLD responses related to the inducer regions go down compared to BOLD responses in a control configuration that did not elicit an illusory shape percept (Figure 1B). Conversely, BOLD responses in the illusory shape regions increase compared to BOLD responses in the control configuration.

The increased BOLD response observed for the illusory shape region compared to the control region is well-known from various previous studies, related not only to illusory figure perception (Lee & Nguyen, 2001), but also figure–ground segmentation (Lamme, 1995) and contour detection (Altmann, Bülthoff, & Kourtzi, 2003) using neuroimaging as well as neurophysiological methods. Instead of relying on predictive coding, this increase in figure-related activity is commonly explained by higher level visual areas detecting the shape and sending excitatory feedback signals to early visual cortex to signal the to be grouped features. According to Kok and de Lange (2014), the suppressive effects related to the inducers are less readily explained by these top-down excitatory feedback modulations. Therefore, they argue that predictive coding provides a coherent explanation for both the enhancing and suppressive effects.

Although it is appealing to frame these results in the context of the visual system as a hierarchical generative model, there might be an alternative explanation for the observed decrease in inducer-related BOLD responses. That is, on each trial, observers were presented either with an illusory shape or a control configuration and this stimulus was presented for 14.4 s in an alternating sequence with four full black disks (presented on the inducer locations, see Figure 1C^1^). Depending on the task, participants had to detect a diamond stimulus that was presented at a random time point in this alternating sequence (“figure task”) or a target letter presented in a rapid letter stream outside the illusory shape area (“letter task”). The potential problem with this alternating sequence of pacmen inducers and full black disks is that, *perceptually*, the illusory shape stimulus consists of a modally completed shape on top of amodally completed *disks.* So, *perceptually*, in this alternating sequence, the inducers do not change, but are continuously present throughout the trial and perceived as four full black disks on which a shape is being presented periodically. Therefore, the observed decrease in inducer-related BOLD response might also be explained by neural adaptation to perceptually stable input in the illusory shape condition. Indeed, in the control condition, the inducers do perceptually change over the course of a trial, yielding the observed difference in BOLD responses. It should be noted that neural adaption in fMRI studies (also known as repetition suppression) is usually observed for repeatedly or continuously presenting the same, physical stimulus (Grill-Spector, Henson, & Martin, 2006; Krekelberg, Boynton, & van Wezel, 2006). A different situation arises in this study however, in which the trial sequence consists of rapidly alternating, physically different stimuli. As argued above, the reasoning is that, if participants perceive the inducers to be continuously present as amodally completed disks, the physical alternation is not perceived as such and neural adaptation to the perceived disks might accumulate over the course of a full trial and result in a decrease of inducer-related activity in the illusory condition compared to the control condition. Interestingly, this explanation in terms of neural adaptation to perceptually stable input implies that neural adaptation in early visual cortex can be modulated by perceived as well as physical input. This might not be surprising given the fact that studies already have shown the changes in V1 BOLD response correspond with perceived rather than actual stimulus size (Murray, Boyaci, & Kersten, 2006).

Although neural adaptation can be invoked as a potential alternative explanation for the decrease in inducer-related BOLD responses, it is important to keep in mind that the observed decrease (∼0.05% signal change difference) is substantially smaller than what would be expected based on classical observations in fMRI adaptation studies (∼1–2% signal change; Grill-Spector et al., 1999; Tootell et al., 1998). It is however hard to exactly predict the size of the expected adaptation effect. That is, there are some factors (albeit speculative) that could obscure a larger adaptation effect. For example, although the neural adaptation argument relies on the potential perceptual consequence of the alternating stimulus sequence throughout a trial, the trial sequence still consisted of physically alternating stimuli rather than constant or repeating stimuli which are traditionally used in fMRI adaptation studies. Furthermore, participants might not have always perceived the amodally completed disks on each trial. Third, the size of fMRI adaptation effects have been shown to be sensitive to the time-scale of the presentation (Krekelberg et al., 2006). Lastly, in both conditions, the trial sequence consists of a perceptually predictable sequence of two alternating frames. Given that it has been shown that expectation about the upcoming stimulus can affect fMRI adaptation effects (Summerfield, Trittschuh, Monti, Mesulam, & Egner, 2008), this expectation effect might interact with the adaptation effect related to the perceptually stable input in the illusory shape condition.

In sum, Kok and de Lange (2014) provide an interesting and fresh take on suppressive and enhancing effects of neural activity in early visual cortex based on the predictive coding framework. Although the enhancement of activity related to the illusory shape fits well with predictive coding, the suppressive effects related to the inducers might also be explained by neural adaptation to perceived input. That is, if the alternating sequence in the illusory shape condition is perceived as a triangle being periodically flashed on top of three full disks, this might lead to neural adaptation to the disks, although the specific contribution of this neural adaptation effect to the data at hand remains unknown. Therefore, the results do not necessarily provide unequivocal evidence for invoking predictive coding as a general explanation for the observed increases and decreases in BOLD responses in early visual cortex.

## Footnotes

1 See https://perswww.kuleuven.be/~u0072929/IllusoryShape_animation.gif and https://perswww.kuleuven.be/~u0072929/ControlConfiguration_animation.gif for animated versions of the trial sequence.
